# End-of-life healthcare use and associated costs for First Nations Australians diagnosed with cancer in Queensland, Australia

**DOI:** 10.1007/s00520-025-09725-x

**Published:** 2025-07-08

**Authors:** Shafkat Jahan, Daniel Lindsay, Abbey Diaz, Ming Li, Kalinda Griffiths, Ian Olver, Gail Garvey

**Affiliations:** 1 First Nations Cancer and Wellbeing Research Program, School of Public Health, Faculty of Health, Medicine and Behavioural Sciences, The University of Queensland, Brisbane, QLD Australia; 2 Cancer Health Economics, Cancer Council Queensland, Brisbane, QLD Australia; 3 The National Centre for Aboriginal and Torres Strait Islander Wellbeing Research, The Australian National University, Australian Capital Territory, Canberra, Australia; 4 Poche SA+NT College of Medicine and Public Health, Flinders University, Darwin, Australia; 5 School of Public Health, The University of Adelaide, Adelaide, Australia

**Keywords:** First Nations Australians, End-of-life care, Health care utilisation, Palliative care, Cancer costs

## Abstract

**Purpose:**

Cancer significantly impacts First Nations Australians, with higher incidence and lower survival rates. However, understanding of end-of-life (EOL) service use and costs in this population is limited. We aimed to assess EOL healthcare utilisation and costs for First Nations cancer patients in Queensland, Australia.

**Methods:**

Retrospective data from CancerCostMod, a linked administrative dataset of all cancer diagnoses in Queensland, were used. This dataset includes records from the Queensland Cancer Registry (QCR) from July 1, 2011, to June 30, 2015, linked to Queensland Health Admitted Patient Data Collection (QHAPDC), Emergency Department (ED) Information Systems, Medicare Benefits Schedule (MBS), and Pharmaceutical Benefits Scheme (PBS) data from July 2011 to June 30, 2018. All diagnosed cancer patients who had died during the study period (*N* = 467) were included. Health service usage and costs during the last 6 months of life were described and compared across care type, comorbidity status, age group, and residential remoteness using Mann–Whitney and Kruskal–Wallis tests.

**Results:**

Individuals had at least one hospital episode (100%), ED visit (83%), MBS claim (96%), and PBS claim (96%). The median overall cost per person for hospital episodes was AUD$40,996, with higher costs for those receiving palliative care (AUD$43,521) and chemotherapy (AUD$50,437) compared to those who did not receive these services (palliative: AUD$34,208, chemotherapy: AUD$38,557). Having comorbidities and living in regional and remote areas were associated with higher hospital costs.

**Conclusion:**

The study findings may guide the re-design and delivery of optimal and culturally appropriate EOL care for First Nations Australians diagnosed with cancer.

**Supplementary Information:**

The online version contains supplementary material available at 10.1007/s00520-025-09725-x.

## Introduction

The global burden of cancer on healthcare systems is increasing due to a growing incidence and improved survival outcomes. This has led to an augmented demand for healthcare services that extend beyond the realm of cancer treatment, incurring greater healthcare costs for both governments and individuals [1–3]. The end-of-life (EOL) period, typically defined as the final 6–12 months before mortality [4], presents a distinct set of healthcare demands that are resource-intensive and expensive [4–7]. The complexities associated with providing comprehensive EOL care pose intricate challenges, both in terms of medical attention and economic considerations.

In Australia, the average excess healthcare costs per cancer patient (costs directly attributable to cancer during the EOL phase) were estimated at AUD$49,733, with hospital admissions accounting for 79% of all costs [6]. This greatly exceeds the average excess costs reported in the initial (first 12 months) and continuing phases of cancer diagnosis (AUD$28,719 and $4,474, respectively). Similar patterns of increased service use and costs during EOL have been observed internationally [7, 8]. A systematic review linked increased EOL cancer care costs with comorbidity, younger age, and race/ethnicity [4]. Further, a US study suggests that minority populations utilise EOL cancer care less than white populations [4]. Although not specific to EOL care, we reported similar findings in Australia, indicating that healthcare costs for Aboriginal and Torres Strait Islander peoples, the First Nations peoples of Australia, are lower than for other Australians owing to lower health-service utilisation [9].

In Australia, guidelines on Optimal Care Pathways for Aboriginal and Torres Strait Islander people emphasise the importance of EOL care [10]. However, there are currently no comprehensive data to guide the (re)design and delivery of optimal and culturally safe EOL care for First Nations Australians diagnosed with cancer. This is despite cancer being the leading burden of disease in this population [11] with higher, and increasing, rates of incidence/mortality compared with other Australians [12].

Generally, the patterns of service use and costs for EOL cancer patients are becoming better understood [4, 7]; however, less is known regarding those for First Nations Australians. A small study (*N* = 58) from Queensland, Australia, has addressed EOL health-service use by First Nations Australians [13]. Participants had a median of five (range 1–27) hospital admissions during this phase, divided across acute (85%) and palliative (15%) care. Most were admitted to hospital via emergency (38%) or outpatient (31%) departments, and admissions were commonly related to cancer (39%) or its treatment (chemotherapy/immunotherapy, 18%). A large proportion of the participants died in hospital (78%) [13]. A larger study from Australia investigated the unmet supportive care needs of First Nations people (*N* = 248) reported 22% of participants had moderate to high levels of unmet need associated with financial concerns [14].

No published data exist on non-hospital outpatient healthcare use and associated costs, including primary and specialist services, and pharmaceuticals, during the EOL phase among First Nations Australians with cancer [15]. As their experiences may differ from other populations, there is a critical need for evidence encompassing hospital admissions and ED presentations, Medicare Benefits Schedule (MBS) and Pharmaceutical Benefits Scheme (PBS) service use, and associated costs related to cancer. The study is aimed at examining patterns of healthcare utilisation during the final 6 months of life for First Nations Australians in Queensland diagnosed with cancer. Utilising linked administrative data, we explored the frequency of hospital admissions, ED visits, MBS and PBS claims, and the associated health system costs and out-of-pocket (OOP) expenses. Our findings may inform future efforts to address these gaps, which could potentially enhance EOL cancer care practices and policies for Indigenous populations globally.

## Methods

### Study design and population

We employed a retrospective cohort design using CancerCostMod [16], a linked administrative dataset containing all cancer diagnoses reported in the Queensland Cancer Registry (QCR), from 1 st July 2011 to 30th June 2015 (*N* = 106,571), excluding non-melanoma skin cancers. The information obtained from the QCR includes patient demographics at diagnosis, cancer type, and mortality data (i.e., date of death). All QCR records were linked to Queensland Health Admitted Patient Data Collection (QHAPDC), Emergency Department Information Systems (EDISs), Medicare Benefits Schedule** (**MBS), and Pharmaceutical Benefits Scheme (PBS) data from 1 st July 2011 to 30th June 2018. Initially, data from 1 st July 2011 to 30th June 2012 (QCR) were linked to July 2011 to June 30, 2015 (other datasets). However, subsequent ethical approval extended the timeframe until June 2015 (QCR) and further linked to other datasets until June 2018, increasing our sample size [17].

We restricted our analysis to individuals identified in the QCR as First Nations Australians ≥ 18 years old at the time of their primary cancer diagnosis (all cancer types included) (*n* = 1849), who survived at least 6 months after diagnosis but died during the study period and had at least one hospital admission (*n* = 467). These inclusion criteria ensured a more stable observation period for meaningful EOL cost analysis. All individuals who met these criteria were included, regardless of whether their cancer was in remission, cured, or if their cause of death was unrelated to cancer. This cohort was linked to other health datasets (EDIS, MBS, and PBS) to assess health service use and related costs (see Supplementary Fig. 1 for cohort development and data linkage process).

We excluded individuals who survived less than 6 months (*n* = 332) as their shorter survival time may introduce variability in service use patterns and skewed cost estimates, which could bias the overall findings. Their healthcare use and costs are summarised in Supplementary Tables 1–2. Subgroup analysis was not feasible due to the small sample size in some of these groups.

### EOL phase

We defined the EOL phase as the 6 months prior to and including the month of death. As dates provided in the QHAPDC dataset only included month and year, the EOL phase for each individual cohort member ranged between 6 and 7 months before death. Due to this lack of specificity, this study does not disaggregate outcomes by month.

### Data sources and key variables

#### Hospital episodes

The QHAPDC dataset covers admissions to public and private Queensland hospitals, with separate records for each episode of care within each admission. These episodes may be new admissions (e.g., patients arriving from home or via the ED) or statistical admissions (e.g., inter-facility transfers). We included all hospital-based episodes during the EOL phase, irrespective of their duration. We quantified episode of care for all types, including acute and palliative, using the care-type indicator contained within each record. Given previous research indicating high chemotherapy use during EOL phase [13], we identified chemotherapy-related episodes using the Australian Refined Diagnostic-Related Group (DRG) of R63Z, capturing only hospital-administered infusions, not those administered in other settings (i.e., day procedure centres).

#### ED visits

The EDIS dataset captures all ED visits for the CancerCostMod cohort, regardless of the reason for the visit. Only ED visits during the EOL phase for the sample of interest were included.

#### MBS and PBS items

All MBS and PBS claims during the EOL phase for the sample were included. These datasets provide comprehensive details for each MBS service and PBS prescription accessed by individuals including date, item code, full charge, government rebate (if applicable), and patient co-payment.

### Assigning costs

Australia’s universal healthcare system offers free public hospital care and subsidised primary healthcare through Medicare, with private health insurance (PHI) optional. Medicare provides free access to public hospital services and subsidised or free primary healthcare outside of hospitals. Hospitals are funded through a combination of federal and state government contributions, PHI and OOP payments. Over 90% of public and around 30% of private hospital funding comes from Governments; PHI covers around half of private hospital costs, with individuals contributing just over 10% [18].

In this study, health system costs include all expenses borne by public and private health services and funders for delivering care. Cost assignment for healthcare episodes in the CancerCostMod dataset is detailed elsewhere [19]. Briefly, each hospital episode-of-care is assigned an Australian Refined DRG code, and each ED visit is given an Urgency Related Group code. These codes are used to determine each healthcare episode costs based on the National Hospital Cost Data Collection report for the given year [20, 21]. OOP costs for each MBS and PBS service were calculated as the ‘gap’ between the total charge and the government rebate. OOP costs for MBS and PBS services were calculated and reported separately. All costs were calculated in Australian Dollars (AUD) and adjusted to the 2020–21 financial year to ensure comparability and account for inflation [22].

### Co-variates

Age and gender information was obtained from QCR. Remoteness, determined by postcode at diagnosis, was classified using the Australian Statistical Geography Standard (ASGS), considering population size and distance to major cities [23]. Participants were classified into metropolitan, regional (inner and outer regional), or remote (remote and very remote). Charlson Comorbidity Index (CCI) scores were calculated using ICD-10-AM codes as the primary reason for hospital episodes during any cancer journey stage [24]. Given our interest in co-occurring conditions with cancer (the index condition of all cohort members), CCI scores were adapted to exclude cancer. Cancer staging data were unavailable for inclusion.

### Statistical analysis

We used descriptive analyses to measure demographic characteristics at the time of diagnosis and to quantify the level of healthcare use by this sample in the final 6 months of life. We calculated the total number of healthcare episodes among the cohort and the median number per person for admitted patient episodes, ED visits, MBS services used, and PBS claims. We used the median as the primary measure for cost and service use due to the non-normal distribution of data and small sample sizes across groups. Descriptive statistics also summarised hospital episodes, including care type, referral source, episode outcome, and reason for care (classified by Major Diagnostic Category).

We quantified the costs to the government from hospital care, ED visits, MBS claims, and PBS prescriptions during the EOL period. However, individual costs were only assessed for MBS and PBS claims, as hospital admissions and ED visits are primarily covered by Medicare or PHI, resulting in no or minimal OOP expenses. We presented total costs and median costs per person for the entire period. Differences in median costs and service use based on chemotherapy or palliative care use, presence of a comorbidity (no, 1 or more), age at diagnosis (18–44 years, 45–64 years, 65 + years), and rurality (metropolitan, regional, and remote) were then examined using Mann–Whitney and Kruskal–Wallis tests, with statistical significance set at *p* < 0.05. We did not perform multivariate analyses due to low numbers in some groups. Analysis was conducted using SAS software [25].

## Results

The demographic characteristics of the 467 individuals in the cohort are presented in Table 1. The mean age at diagnosis was 60.9 (SD = 14.3) years. Of the 467 deaths, 411 (88%) were due to cancer with lung cancer being the most common and over 50% had a comorbidity.

**Table 1:** Demographic characteristics at time of cancer diagnosis for First Nations Australians who survived at least 6 months after diagnosis but died during 2011–2015

Characteristics (*N* = 467)	*n* (%)
**Age at diagnosis**	
18–44 45–64 65 +	83 (18) 189 (40) 195 (42)
**Sex**	
Male Female	247 (53) 220 (47)
Remoteness^a^	
Metropolitan Regional (inner and outer) Remote (remote and very remote) Missing	102 (22) 225 (48) 129 (28) 11 (2)
**Cancer type**	
Lung Digestive Colorectal Head and neck Gynaecological Breast Other	122 (26) 86 (18) 42 (9) 31 (7) 30 (6) 29 (6) 127 (28)
CCI score^b^	
0 1–2 3 +	200 (43) 111 (24) 145 (33)
**Year of diagnosis**	
2011–12 2012–13 2013–14 2014–15	121 (26) 119 (25) 108 (23) 119 (26)

^a^Postcode not reported for 2% within the dataset. Remoteness categories are based on ASGS ^23^

^b^Charlson Comorbidity Index ^24^

### Hospital episodes

All cases had at least one hospital admission (median 5, range 1–49) (Table 2). Most episodes (86%) occurred in public hospitals, with primary referrals from the ED (42%), outpatient department (18%), and routine readmissions without referrals (16%). Individuals reported having no hospital insurance for 82% of all hospital episodes. Although 72% of hospital episodes ended in discharge to home/usual residence, most cases (*n* = 365, 78%) died in hospital. The leading reasons for admission included myeloproliferative disorders and other neoplasms (25%), respiratory system diseases (16%), and digestive system disorders (13%).

**Table 2:** Healthcare service use during the EOL phase for First Nations Australians with cancer (*N* = 467)

Healthcare service	Total	Median (IQR) per person	Range	*n* (%) of sample using service during EOL
Hospital episodes	3028	5 (3–8)	1–49	467 (100)
Public patient^a^	2591 (86%)	5 (3–8)	0–43	430 (92)
Private patient^a^	437 (14%)	8 (3–12)	0–49	48 (10)
Acute care^b^	2572 (85%)	4 (2–7)	0–48	445 (95)
Palliative care^b^	420 (14%)	1 (1–2)	0–14	260 (56)
Hospital-based chemotherapy^c^	560 (19%)	5 (2–8)	0–19	97 (21)
ED visits	1479	3 (1–5)	0–40	389 (83)
MBS services^d^	31,014	56 (30–91)	0–908	447 (96)
PBS claims	22,881	46 (29–70)	0–275	447(96)

^a^*n*, % of all hospital episodes

^b^Hospital-based acute and palliative care (*n*, % of all hospital episodes). Other care type classifications include geriatric, mental, psychiatric, rehabilitation, and newborn

^c^*n*, % of all episodes. Note this does not cover all possible cases of chemotherapy potentially used by participants, only those in the hospital setting

^d^Medicare-subsidised medical services, including doctor visits, tests, and procedures

### Other healthcare use

83% of the cohort had at least one ED visit during the EOL (median 3, range 0–40); approximately half had three or more ED visits during this time. Most of the individuals had at least one MBS or PBS claims (96% for both) (Table 2).

### Costs during EOL

The median cost per person during the EOL period were highest for hospital episodes ($40,996; IQR $22,565–$70,973), followed by ED presentations ($2629; IQR $1339–$4166), MBS services ($2462; IQR $1340–$4737), and PBS claims ($2188; IQR $852–$5244) (Table 3). Hospital-based palliative care episodes accounted for 18% of all hospital costs with a median cost per person of $12,778 (IQR = $7300–$22,722). Total OOP costs to individuals during EOL care incurred through MBS and PBS claims were just over $100,000 (median $114; IQR $36–249), primarily driven by PBS claims (84%).

**Table 3:** Costs incurred by healthcare funders and individuals during the EOL phase for First Nations Australians with cancer (*N* = 467)

Cost incurred by	Healthcare system	Total cost (AUD$)^a^	Median (IQR) per person (AUD$)	Range (AUD$)
Government	Hospital	25,067,642	40,996 (22,565–70,973)	911–498,340
	ED	1,242,030	2629 (1339–4166)	0–18,290
	MBS	1,752,614	2462 (1340–4737)	37–34,263
	PBS	2,482,829	2188 (852–5244)	0–122,916
Individual^b^	MBS	16,432	0	0–3632
	PBS	86,426	103 (36–249)	0–1920

^a^*AUD* Australian dollar, *IQR* interquartile range

^b^OOP costs to individuals refer to the direct expenses incurred for healthcare services that are not reimbursed by Medicare or private health insurance

### EOL health service use and costs based on various factors

The median health service use and costs per person during the EOL phase for based on factors of interest are presented in Tables 4 and 5, respectively. The MBS OOP costs are not shown in Table 5 as costs by sub-group were negligible (no OOP costs for 95% individuals).

**Table 4:** Median (IQR) number of healthcare services used during the EOL phase for First Nations Australians with cancer based on various factors (*N* = 467)

Factor	*n*	Hospital episodes	ED visits	MBS claims	PBS claims
Palliative care^b^					
Yes No	260	5.5 (3–9)*	3 (2–5)	61 (34–96)*	49 (32–71)*
	207	4 (2–7)	3 (1–5)	46 (26–83)	42 (25–68)
Chemotherapy^c^					
Yes No	97	12 (8–16)*	3 (2–5)	83 (53–126)*	54 (40–85)*
	370	4 (2–6)	3 (1–5)	45 (27–82)	43 (27–66)
Comorbidity^d^					
0 1 or more	200	4 (2–8)	2 (1–5)	54 (29–96)	43 (24–66)
	267	6(3–9)*	3 (2–5)	57 (30–84)	50 (33–72)*
Age at diagnosis					
18–49 50–64 65 +	83	7 (4–9)*	3 (2–5)	55 (32–86)	45 (27–71)
	189	5 (3–9)	3 (2–5)	61 (29–92)	45 (27–70)
	195	4 (2–7)	2 (1–4)	48 (28–91)	47 (29–70)
Remoteness					
Metropolitan Regional Remote	102	6 (3–10)	3 (1–4)	70 (40–104)*	53 (36–74)^a^
	225	5 (3–8)	3 (2–5)	57 (30–97)	49 (31–72)
	129	5 (3–8)	2 (1–5)	45 (25–70)	36 (21–54)

*Statistical significance at *p* < 0.05

^a^Used hospital-based palliative care

^b^Used in hospital chemotherapy

^c^CCI score

**Table 5:** Median (IQR) per person costs during the EOL phase for First Nations Australians with cancer based on various factors (*N* = 467)

Factor	*n*	Hospital costs ($AUD)	ED costs ($AUD)	PBS OOP costs ($AUD)
Palliative care^b^				
Yes No	260	43,521 (26,476–71,653)*	2513 (1586–4062)	119 (43–270)*
	207	34,208 (16,817–66,109)	2649 (1263–4349)	92 (25–231)
Chemotherapy^c^				
Yes No	97	50,437 (30,224–84,010)*	3150 (1639–4416)	195 (85–348)*
	370	38,557 (20,963–64,321)	2508 (1277–4105)	91.5 (25–218)
Comorbidity^d^				
0 1 or more	200	32,065 (15,201–56,631)	2006 (989–3548)	112 (40–347)
	267	46,977 (29,108–79,209)*	3086 (1730–4429)*	102 (34–267)
Age at diagnosis				
18–49 50–64 65 +	83	53,389 (32,393–95,092)*	3368 (1358–4619)*	162 (71–348)*
	189	43,538 (24,458–71,951)	2930 (1734–4374)	115 (38–269)
	195	32,283 (17,233–57,791)	2116 (1254–3632)	89 (24–208)
Remoteness				
Metropolitan Regional Remote	102 225 129	39,930 (23,996–63,885)* 39,171 (21,684–69,902) 46,187 (23,829–74,994)	2996 (1358–4062) 2781 (1641–4419) 2285 (980–3895)	168 (9–300) 94 (30–236) 98 (39–233)

*Statistical significance

^a^Used hospital-based palliative care

^b^Used in patient chemotherapy

^c^CCI score

Service use and costs varied across groups. Individuals who used hospital-based palliative care and chemotherapy had significantly more hospital episodes, MBS and PBS claims and a greater median hospital and PBS costs than those who did not use these services (*p* < 0.05).

Those with at least one comorbidity had significantly more hospital episodes and PBS claims and incurred significantly higher costs to the government through hospital episodes and ED visits than those without comorbidity (*p* < 0.05). Younger individuals (18–49 years) had significantly more hospital episodes and incurred a greater cost to the government (though hospital and ED costs) than older individuals (*p* < 0.05). Additionally, PBS OOP costs were notably lower for those aged 65 + compared with those aged 18–49 years (*p* < 0.05).

There was no significant difference in hospital episodes or ED visits, and individuals in remote areas utilised significantly higher MBS and PBS services than those in regional and metropolitan areas (*p* < 0.05). In contrast, individuals from rural areas had higher hospital episode costs but lower ED visit costs and PBS claims compared with those from metropolitan areas (*p*’s < 0.05).

Health service use patterns were higher among those surviving ≥ 6 months, with more hospital episodes (5 vs. 3), palliative care use (54% vs. 42%), and chemotherapy (19% vs. 2%) (S Table 1). Consequently, median costs per person were higher in this group compared to those who survived < 6 months (S Table 2).

## Discussion

Our study bridges the knowledge gap regarding patterns in healthcare-service use and costs in the EOL phase for First Nations Australians diagnosed with cancer. The findings reveal significant healthcare costs incurred by the government, primarily due to admitted-patient hospital care, consistent with previous research [4, 5]. Significant pharmaceutical OOP costs to individuals were observed. Higher health care use and associated costs were observed among younger individuals, those with comorbidities, and rural residents, underscoring the need for tailored support and resource allocation in EOL cancer care for these segments of the First Nations population.

The median hospitalisation cost in the EOL period was $40,996, consistent with earlier Australian studies reporting inpatient costs between $49,733 and $55,357 in the final year of life [6, 26]. Hospital episodes accounted for 82.1% of EOL costs in our cohort, which is similar to that reported by Goldsbury et al. for non-Indigenous patients (76%) [6]. We observed lower costs for prescription medicines (5.7% vs. 11%) and MBS services (8.1% vs. 10%) and slightly higher costs for ED presentations (4.1% vs. 3%). Differences may reflect variations in study timeframe (6 vs. 12 months), population (First Nations vs. general), cohort characteristics, and statistical approaches.

The higher number of public hospital episodes observed in our study may reflect low PHI. Even where PHI exists, limited private healthcare options in regional areas may reduce access, increasing reliance on public hospitals. Almost all individuals had at least one MBS or PBS claim, and nearly half had three or more ED visits. Although most patients had at least one acute hospital episode, only just over half received hospital-based palliative care, and one-fifth had chemotherapy during the EOL phase.

Median EOL care OOP costs per person were lower in this study than those reported for First Nations Australians in the first year following diagnosis (AUD$103 vs. AUD$177) [27]. MBS OOP costs were minimal, with over 75% reporting no costs despite high utilisation, suggesting adequate Medicare coverage. However, it is also possible that individuals, aware of their limited time, choose to avoid costly healthcare due to potential OOP expenses. As pharmaceutical claims constituted most OOP costs, expanding access to medications through programmes like the Closing the Gap initiative [28] may help reduce financial burden.

Healthcare service utilisation and costs varied in this sample based on hospital-based palliative care and chemotherapy utilisation, age at diagnosis, and rurality [29]. Approximately one-quarter of individuals received at least one in-hospital chemotherapy, consistent with previous findings for First Nations Australians with cancer [13, 29]. Higher costs observed among these patients may reflect greater care complexity rather than the direct costs of chemotherapy itself. This highlights the importance of aligning treatment decisions with patients’ goals and cultural preferences [30].

We identified a similar level of hospital-based palliative care use among First Nations Australians as reported in Australia [31], where just over half of the general patient population received such care. Despite the known benefits of palliative care in cancer treatment [32, 33], its utilisation remains low in this population, emphasising the need for strategies to increase its adoption. General practitioners (GPs) play a key role in referrals [34], yet cultural misunderstandings among health care professionals and limited awareness among First Nations peoples may hinder engagement with palliative services [35]. We observed higher healthcare service use costs (except ED visits) for those receiving hospital-based palliative care compared to those not receiving hospital-based palliative care, which may suggest a greater financial burden that may deter its use. However, we could not assess whether palliative care caused this increase nor did we capture care provided outside hospitals. This gap highlights the need for future research exploring community-based palliative care for First Nations Australians.

Over half of the cohort had at least one comorbidity, contributing to higher service use and costs. Interestingly, only a quarter of hospital episodes were cancer-related, suggesting that care for other conditions places a larger burden on the system. Given the greater multimorbidity and poorer outcomes among First Nations people, managing non-cancer conditions is critical during EOL [36, 37]. Multidisciplinary care models that address both cancer and comorbidities may improve health and cost outcomes.

Our findings also support previous evidence of disparities in healthcare use among cancer patients based on rurality [29]. Rural patients had fewer ED visits, hospital episodes, and MBS/PBS claims. Higher hospital admissions among First Nations peoples with cancer living in more rural areas may reflect more complex cases and higher costs, consistent with the high comorbidity rates in the sample.

The cultural context shaping how First Nations Australians perceive healthcare, especially at EOL, is crucial for interpreting this study’s results. Many wish to die ‘on Country’ (i.e., traditional lands) [10, 38, 39] but have limited access to culturally safe and community-based care, especially in rural settings. However, 78% of our cohort died in hospital, possibly indicating challenges in accessing community-based palliative care in rural settings. Culturally safe EOL care models are essential and must allow individuals to maintain cultural and spiritual connecting without discrimination [40]. A deep-rooted mistrust of the healthcare system among First Nations Australians [39, 41] may contribute to the avoidance of care that is perceived as culturally inappropriate.

Utilising administratively linked data, we provide a comprehensive overview of health service use and related costs, but some limitations must be acknowledged. Indirect OOP costs (e.g., travel and accommodation) substantial for remote patients [42, 43] were not captured, likely underestimating total financial burdens. The lack of precise admission dates prevented accurate cost quantification for specific months leading up to death, leaving unclear when healthcare utilisation and costs peaked during the EOL. Limited sample size and the low proportion of chemotherapy-related hospital episodes (21%) restricted reliable estimation of leading admission causes, including chemotherapy complications, i.e., neutropenic sepsis. Additionally, as the analysis included only patients with at least one hospital episode and survived ≥ 6 months post-diagnosis, individuals who used only ED, MBS, or PBS services were not captured, potentially underestimating overall health service use and costs. However, this likely had minimal impact, as only 4.3% had no hospital episodes. Finally, as costs were assigned using DRG codes that bundle all services within a hospital episode, chemotherapy and palliative care, being components of hospital care, directly contribute to the outcomes. This makes the grouping variables and outcomes interdependent, meaning the independence assumption underlying the Mann–Whitney U tests is not met. Therefore, these comparisons should be interpreted as descriptive and exploratory, aimed at identifying patterns in healthcare use and costs rather than establishing causal or independent group effects*.*

Our study is the first to quantify healthcare use and costs in the final 6 months of life for First Nations Australians with cancer using linked administrative health data. The findings highlight the substantial burden on healthcare systems during this period and underscore the need for culturally competent EOL care that reflects the specific needs and preferences of First Nations peoples. Enhancing culturally appropriate EOL care for First Nations Australians is a crucial step to addressing healthcare inequities, fostering trust, and ensuring care delivery aligns with cultural values, spiritual practices, and community expectations. These insights could inform health policies in Australia and other countries with Indigenous populations facing similar challenges.

Understanding health service use and cost patterns might support clinicians in informed decision-making and advocacy while guiding health services and policymakers in identifying gaps, effectively allocating resources, and designing targeted interventions. Further research and tailored interventions are needed to effectively address the unique EOL needs of this population.

## Supplementary Information

Below is the link to the electronic supplementary material.

ESM 1 (DOCX 128 KB)

ESM 2 (DOCX 24.0 KB)

## Data Availability

The data that support the findings of this study are not openly available due to reasons of sensitivity. The data is securely stored in the"SURE"server maintained by Sax Institute. https://www.saxinstitute.org.au.

## Code Availability

Due to privacy constraints associated with our ethics approval, the datasets and coding used in the current study cannot be made publicly available. Our ethics approval explicitly prohibits the sharing of data and code.
