# Supplementary figures and images for: End-of-life healthcare use and associated costs for First Nations Australians diagnosed with cancer in Queensland, Australia
Source: Support Care Cancer. 2025 Jul 8;33(8):669. doi: 10.1007/s00520-025-09725-x (PMC12238182; doi:10.1007/s00520-025-09725-x)

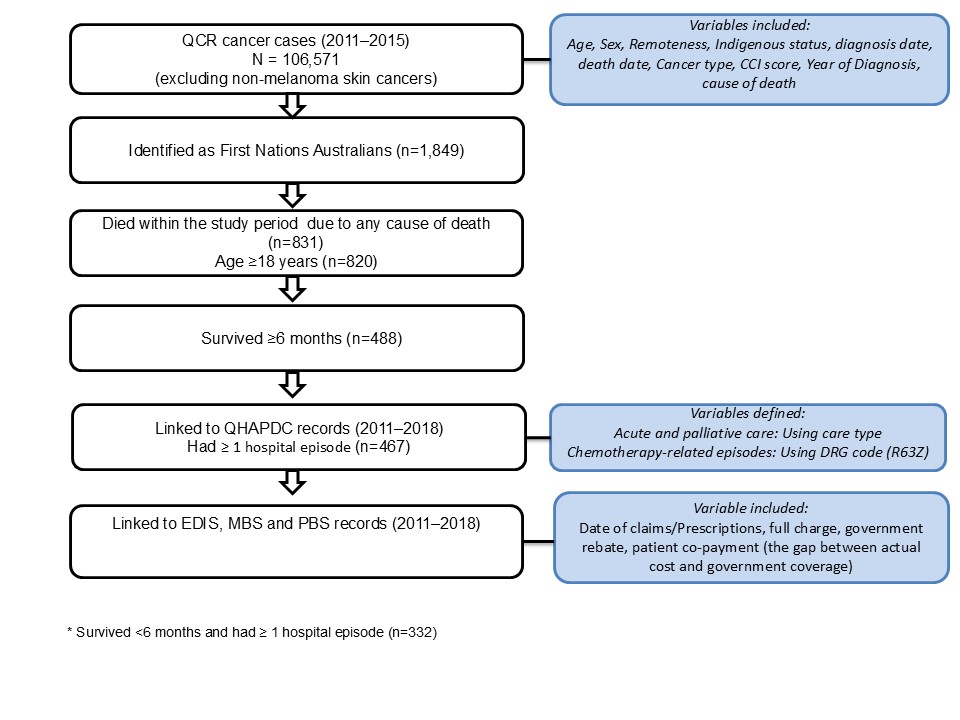
Supplementary Figure 1: Data linkage and cohort selection process.

Supplement: Supplementary file 1 — (DOCX 128 KB) [file 520_2025_9725_MOESM1_ESM.docx]
