# Supplementary material for: End-of-life healthcare use and associated costs for First Nations Australians diagnosed with cancer in Queensland, Australia
Source: Support Care Cancer. 2025 Jul 8;33(8):669. doi: 10.1007/s00520-025-09725-x (PMC12238182; doi:10.1007/s00520-025-09725-x)
Supplement: Supplementary file 2 — (DOCX 24.0 KB) [file 520_2025_9725_MOESM2_ESM.docx]

**S Table 1**. Healthcare service use during the EOL phase for First Nations Australians with cancer who survived < six months (N=332)

| Healthcare service | Total | Median (IQR) per person | Range | n(%) of sample using service  during EOL |
| --- | --- | --- | --- | --- |
| *Hospital episodes* | 1141 | 3(2–5) | 1–22 | 332 (100) |
| Public patient^a^ | 1111 (97.3%) | 3 (2–5) | 1–22 | 319 (96.1) |
| Private patient^a^ | 30 (2.7%) | 2(1–2) | 1–5 | 13 (3.9) |
|  |  |  |  |  |
| Acute care^b^ | 1126 (99%) | 3 (2–4) | 1–14 | 295 (88.8) |
| Palliative care^b^ | 614 (54%) | 1 (1–2) | 1–8 | 138 (41.6) |
|  |  |  |  |  |
| Hospital–based chemotherapy^c^ | 23 (2%) | 1(1–3) | 1–7 | 10 (3) |
|  | | | | |
| ED visits | 657 | 2 (1–3) | 0–13 | 251 (75.6) |
| MBS services^d^ | 7583 | 17 (8–34) | 1–234 | 280 (84.3) |
| PBS claims | 5418 | 13 (6–27) | 1–108 | 1. (81) |

1. n, % of all hospital episodes.
2. Hospital-based acute and palliative care (n, % of all hospital episodes). Other care type classifications include geriatric, mental, psychiatric, rehabilitation, and newborn.
3. n, % of all episodes. Note this does not cover all possible cases of chemotherapy potentially used by participants, only those in the hospital setting.
4. Medicare-subsidised medical services, including doctor visits, tests, and procedures.

**S Table 2**. Costs incurred by healthcare funders and individuals during the EOL phase who survived < six months for First Nations Australians with cancer (N=332)

| Cost incurred by | Healthcare system | Total cost (AUD$)^a^ | Median (IQR) per person  (AUD$) | Range  (AUD$) |
| --- | --- | --- | --- | --- |
| Government | Hospital | 12,940,532 | 34,905 (18,838–52,002) | 1,496–243,403 |
|  | ED | 573,016 | 1,924 (1,035–2,852) | 0–11,195 |
|  | MBS | 428,589 | 1,723 (845–3,025) | 45–16,271 |
|  | PBS | 307,013 | 363 (95–994) | 0–28,250 |
| Individual^b^ | MBS | 46,417 | 0 (0–0) | 0–13,761 |
|  | PBS | 21,413 | 30 (11–98) | 0–716 |

1. Abbreviations: AUD: Australian dollar; IQR: Interquartile range.
2. OOP costs to individuals refer to the direct expenses incurred for healthcare services that are not reimbursed by Medicare or private health insurance.
